# Factors associated with academic resilience in nursing students: the role of individual, academic, and social factors

**DOI:** 10.1186/s12909-026-08875-8

**Published:** 2026-02-27

**Authors:** Milad Sales Bushehri, Mohammadreza Dinmohammadi, Mohammadjavad Farahani-Hosseinabadi, Mohammad Saatchi

**Affiliations:** 1 ¹Department of Nursing, University of Social Welfare and Rehabilitation Sciences, Tehran, Iran; 2 Determinants of Health Research Center, University of Social Welfare and Rehabilitation Sciences, Tehran, Iran; 3 Department of Biostatistics and Epidemiology, University of Social Welfare and Rehabilitation Sciences, Tehran, Iran

**Keywords:** Academic resilience, Nursing students, Social support, Mental health, Academic burnout

## Abstract

**Background and aim:**

Academic resilience is a critical determinant of progress. Nursing students face significant stress due to both theoretical and clinical challenges. This study aimed to identify the level of academic resilience and its statistically associated factors among nursing students using self-report measures.

**Methods:**

In this correlational study, 375 undergraduate nursing students from three major medical universities in Tehran were selected via stratified random sampling (proportional to strata size) during April-May 2025. Data collection utilized the Academic Resilience Scale (ARS) and a researcher-developed questionnaire (validated via content and face validity). Data were analyzed using descriptive statistics, non-parametric tests, and multiple linear regression, with careful monitoring of residual diagnostics and multicollinearity (VIF).

**Results:**

The students’ mean academic resilience score was 89.89 ± 12.49, indicating a moderate-to-high level. Regression analysis identified seven significant statistical predictors: gender, health status, living situation, satisfaction with major, support resources, nutrition, and non-smoking. These factors collectively explained 19.6% (Adjusted R²) of the variance in resilience.

**Conclusion:**

While several individual and social factors are associated with resilience, the cross-sectional nature of the study limits causal inferences. Interventions focusing on lifestyle and support systems may enhance resilience.

**Supplementary Information:**

The online version contains supplementary material available at 10.1186/s12909-026-08875-8.

## Introduction

Entering university is a pivotal and sensitive stage in the lives of a society’s youth. Along this path, students encounter new challenges stemming from entering a different environment and accepting new responsibilities [1]. Many students lack sufficient familiarity with the university atmosphere; separation from family and the absence of necessary skills for adapting to dormitory life are among the issues that can lead to feelings of loneliness and dissatisfaction with living conditions [2]. Furthermore, confronting theoretical classes and clinical courses, along with numerous assignments and projects during their studies, can impose significant pressure on students [3]. These conditions may lead to a decline in student performance [4, 5]. The stress resulting from these courses and various assessments can cause academic burnout and negatively impact students’ mental health [6]. Academic burnout is a serious and undeniable issue among students, affecting not only their academic performance but also their mental and overall health [7].

Students in health science disciplines, particularly nursing, are required not only to attend theoretical classes but also to gain practical experience in clinical environments. This direct exposure to real treatment settings subjects them to specific stressors arising from academic pressures and clinical encounters [8]. These pressures can lead nursing students to face greater challenges during their education, consequently impacting their academic performance [9]. Due to their direct contact with patients, nursing students who lack sufficient knowledge and skills cannot provide optimal services, which can lead to a reduction in the quality of nursing care [10, 11]. Therefore, students’ efforts to achieve academic success and their ability to cope with the problems and challenges of their education (including low grades, heavy assignments, time constraints, exam stress, and clinical placements) influence their resilience, potentially strengthening or weakening it [12–15].

Academic resilience represents a student’s capacity to confront the challenges and stressors of the educational path, enabling them to successfully overcome these obstacles [16]. Students who possess this capability achieve greater self-efficacy, their academic performance improves, and they attain superior outcomes in their educational journey [17]. Research indicates that these students not only have a better quality of academic life but are also able to control situations and overcome stress [18].

According to existing theories, factors such as younger age, higher GPA, ability in independent learning, social support from family and friends, and allocating time for recreation and leisure are effective in promoting academic resilience. Furthermore, participation in mindfulness-based stress reduction programs is another influential factor [19–23]. Studies show that individual and social factors also affect students’ academic resilience. Particularly among nursing students, this resilience is influenced by a combination of individual and social factors; understanding and identifying these factors is essential for strengthening students’ abilities to face academic challenges and achieve success [24].

Previous research has utilized various methods to measure resilience in students. However, one instrument is the Academic Resilience Scale (ARS), which specifically assesses three dimensions of resilience: perseverance, adaptive help-seeking and reflection, and negative affect and emotional responses. This self-report tool helps students better understand their abilities to cope with academic challenges. In this regard, the ARS plays a significant role as an important analytical tool in identifying students’ individual strengths and weaknesses [25].

Despite international research, there is a lack of critical synthesis regarding how individual, academic, and social variables interact within the Iranian nursing education context. The conceptual framework of this study views academic resilience as a dynamic process influenced by internal and external resources. This framework directs our inquiry into whether demographic traits, lifestyle choices, and social support systems collectively provide a buffer against academic stressors. This study aims to fill the gap by identifying these associations and providing a basis for targeted support mechanisms.

## Methods

This study employed a correlational design to explore associations between variables. The target population comprised all undergraduate nursing students at the medical sciences universities in Tehran (Iran, Tehran, and Shahid Beheshti) during the 2024–2025 academic year.

### Participants and sampling

Three hundred seventy-five nursing students were selected. To ensure representativeness, stratified random sampling was used, with students stratified by academic year and native status. The number of participants from each university was proportional to the total population of nursing students in those institutions.

Inclusion criteria were willingness and consent to participate, being an undergraduate nursing student, having completed at least one academic semester and being familiar with the university environment, and having no diagnosed physical or mental health problems that would affect academic resilience. Exclusion criteria included incomplete questionnaires, visiting or transfer students, and students with extended academic tenure (more than 8 semesters).

### Data collection

Data were collected during April and May 2025. A total of 386 questionnaires were received. After removing incomplete questionnaires, 375 complete questionnaires were entered into the analysis process.

### Instruments

The data collection tool consisted of two parts. The first part was a researcher-developed questionnaire on individual, academic, and social factors (The English language version of the Factors Questionnaire is provided in Supplementary File 1), which was formulated through a review of literature and related studies. Face and content validity were confirmed by a panel of 5 nursing education experts. While this tool provided comprehensive data, we acknowledge that full psychometric testing (e.g., Exploratory or Confirmatory Factor Analysis) was not conducted, which is addressed in the limitations. The second part was the Academic Resilience Scale (ARS) [25]. In this study, the Persian validated version by Vahedi [26] was used, which includes 24 items and 3 subscales: Perseverance (10 items), Adaptive Help-Seeking (9 items), and Negative Affect and Emotional Response (5 items). Scoring was based on a 5-point Likert scale (from 1 = Very Low to 5 = Very High), with 6 items reverse-scored. The total score ranged from 24 to 120, with higher scores indicating greater academic resilience. In this study, the internal consistency (Cronbach’s alpha) of the total instrument was found to be 0.80.

### Ethical considerations

This study was approved by the Institutional Review Board (Code: IR.USWR.REC.1403.225) and adhered to the Declaration of Helsinki. Informed consent was obtained from all participants.

### Data analysis

Data were analyzed using SPSS (v. 27). Due to the non-normal distribution of resilience scores (*p* < 0.05 in K-S test), multiple linear regression (Backward method) was applied. We monitored for multicollinearity using the Variance Inflation Factor (VIF) and evaluated residual diagnostics to ensure model fit. Standardized coefficients (β) were reported to allow for a comparison of the relative strength of the predictors. The significance level for all tests was set at *p* ≤ 0.05.

## Results

Of the 386 questionnaires received, 375 were analyzable (response rate: 97.1%). The mean age of the research units was 21.54 years (SD 2.55), and their mean cumulative GPA was 17.08 (SD 1.12). The mean and standard deviation of the total academic resilience score for nursing students was 89.89 (SD 12.49) (Range: 31 to 118) (Table 1).

**Table 1:** Descriptive indices of academic resilience and its dimensions (*N* = 375)

Variable	Minimum	Maximum	Mean	SD
Academic Resilience (Total Score)	31	118	89.89	12.49
Perseverance	15	49	37.60	5.43
Adaptive Help-Seeking	9	45	34.60	5.13
Negative Affect and Emotional Response	5	25	17.70	4.34

The mean age of students was 21.54 (2.55). Students’ age was not correlated with their academic resilience (*r* = 0.03, *P* = 0.57). Furthermore, the students’ cumulative GPA was 17.08 (1.12) and had no correlation with their academic resilience (*r* = 0.078, *p*=0.134). The gender distribution in the sample was nearly equal (50.9% male), and the majority of students (95.5%) were single. Other descriptive findings are presented in Tables 2, 3 and 4.

**Table 2:** Bivariate analysis of individual factors related to academic resilience (*N* = 375)

Individual Factors		Frequency (Percent)	Mean Rank	Statistic, df	*P* value
Gender	Male	191 (50.9)	201.00	H = 5.60, df = 1	0.018
	Female	184 (49.1)	174.51		
Marital Status	Married	17 (4.5)	206.00	H = 0.49, df = 1	0.483
	Single	358 (95.5)	187.15		
Native Status	Native (Tehran)	202 (53.9)	191.24	H = 0.39, df = 1	0.531
	Non-native (Other cities)	173 (46.1)	184.22		
Living Situation	With family at home	182 (48.5)	191.60	H = 5.69, df = 2	0.058
	Alone	39 (10.4)	220.31		
	Dormitory	154 (41.1)	175.57		
Sleep Status	Hypersomnia	50 (13.3)	151.34	H = 11.90, df = 2	0.003
	Sufficient/Regular sleep	189 (50.4)	205.37		
	Insomnia/Poor sleep	136 (36.3)	177.34		
Exercise	Regularly	75 (20.0)	183.61	H = 0.77, df = 2	0.681
	Occasionally	166 (44.3)	193.50		
	No	134 (35.7)	183.65		
Balanced Nutrition	Regularly	148 (39.5)	208.77	H = 19.85, df = 2	*P* < 0.001
	Occasionally	173 (46.1)	187.70		
	No	54 (14.4)	132.04		
Religious Practices	Regularly	80 (21.3)	215.19	H = 9.48, df = 2	0.009
	Occasionally	140 (37.3)	192.26		
	No	155 (41.3)	170.12		
Smoking/tobacco Use	Regularly	17 (4.5)	132.85	H = 6.70, df = 2	0.035
	Occasionally	59 (15.7)	171.99		
	No	299 (79.7)	194.29		
Social Media Use	Regularly	265 (70.7)	183.91	H = 2.41, df = 2	0.300
	Occasionally	106 (28.3)	199.98		
	No	4 (1.1)	141.50		
Health Status	History of physical illness	40 (10.7)	134.71	H = 15.73, df = 2	*P* < 0.001
	History of mental illness	9 (2.4)	115.56		
	Completely healthy	326 (86.9)	196.54		

**Table 3:** Bivariate analysis of academic factors related to academic resilience (*N* = 375)

Academic Factors		Frequency (Percent)	Mean Rank	Statistic, df	*P* value
Academic Year	First year	105 (28.0)	190.35	H = 0.27, df = 3	0.965
	Second year	103 (27.5)	189.11		
	Third year	92 (24.5)	188.71		
	Fourth year	75 (20.0)	182.31		
Satisfaction with Major	Satisfied	255 (68.0)	204.51	H = 18.51, df = 1	*P* < 0.001
	Not satisfied	120 (32.0)	152.91		
Satisfaction with University	Satisfied	277 (73.9)	200.53	H = 14.17, df = 1	*P* < 0.001
	Not satisfied	98 (26.1)	152.59		
Intend to Continue Education	Yes	227 (60.5)	200.63 168.63	H = 7.82, df = 1	0.005
	No	148 (39.5)			
Considered Dropping Out	Yes	108 (28.8)	201.10	H = 13.54, df = 1	*P* < 0.001
	No	267 (71.2)	155.63		
Intention to Emigrate	Yes	257 (68.5)	195.64	H = 0.85, df = 1	0.355
	No	118 (31.5)	184.49		

**Table 4:** Bivariate analysis of social factors related to academic resilience (*N* = 375)

Social Factors		Frequency (Percent)	Mean Rank	Statistic, df	*P* value
Employment Status	Employed	94 (25.1)	198.88	H = 1.27, df = 1	0.261
	Unemployed	281 (74.9)	184.36		
Income Status	Have income	119 (31.7)	191.65	H = 0.20, df = 1	0.656
	No income	256 (68.3)	186.30		
Peer Relations	Regular	178 (47.5)	197.40	H = 5.68, df = 2	0.059
	Occasional	186 (49.6)	182.82		
	No	11 (2.9)	123.41		
Extracurricular Activity	Regular	59 (15.7)	228.58	H = 26.62, df = 2	*P* < 0.001
	Occasional	179 (47.7)	202.27		
	No	137 (36.5)	151.88		
Support Resources	Regular	215 (57.3)	205.44	H = 13.5, df = 2	0.001
	Occasional	147 (39.2)	166.25		
	No	13 (3.5)	145.46		

Variables that were significant in the bivariate analyses of individual, academic, and social factors were entered into the multiple linear regression model using the Backward method to determine predictors. Multiple linear regression analysis indicated that the model was significant (F = 10.61, *p* < 0.001). The Adjusted R² of 0.196 suggests that the seven identified factors explain approximately 19.6% of the variance in academic resilience. The VIF for all variables was below 2.0, indicating no significant multicollinearity issues.

Multiple linear regression analysis showed that gender (being male), living situation (living alone), balanced nutrition, smoking/tobacco use (non-use), health status (no history of mental illness), satisfaction with major (being satisfied), and possession of support resources (regular) were the significant factors that associated with academic resilience among nursing students (Table 5).

**Table 5:** Parameters of the final regression model for predicting academic resilience (backward method)

Variable	B	S.E.	T	*P*	95% Confidence Interval
(Constant)	75.15	4.850	15.49	*P* < 0.001	(65.61, 84.69)
Gender (Male)	4.61	1.18	3.91	*P* < 0.001	(2.29, 6.93)
Living Situation (Alone)	4.55	2.05	2.22	0.027	(0.52, 8.58)
Nutrition (Regular)	5.12	1.93	2.66	0.008	(1.33, 8.92)
Nutrition (Occasional)	5.23	1.78	2.94	0.004	(1.73, 8.73)
Smoking (Regular)	−8.15	2.89	−2.82	0.005	(−13.84, −2.46)
Health Status (Mental illness)	−13.55	3.82	−3.55	*P* < 0.001	(−21.07, −6.03)
Satisfaction with Major (Satisfied)	5.68	1.25	4.54	*P* < 0.001	(3.22, 8.14)
Support Resources (Regular)	3.91	1.51	2.59	0.010	(0.94, 6.88)

## Discussion

The results indicate a moderate-to-high level of resilience. However, interpretations regarding “predictors” must be made with caution; statistical prediction in this cross-sectional context does not imply causal directionality. This result aligns with numerous international and national studies that consistently report moderate to high resilience levels among nursing students [4, 9, 27–31]. Such a level of resilience is crucial for managing the high academic and clinical stress inherent in nursing [9, 32]. In the dimensional analysis, the highest mean was related to “Perseverance” and the lowest to " Negative Affect and Emotional Response,” suggesting that while students are determined in pursuing their goals, they may be more vulnerable in managing emotional responses to failure—an issue requiring special attention in support programs [25].

The most important finding of this study was the identification of 7 key factors among numerous individual, academic, and social variables that could significantly associated with academic resilience after controlling for other variables.

Regarding gender, the current study’s results indicated that male students possessed higher academic resilience. This finding is consistent with some studies on nursing and other medical science students [33, 34]. Nevertheless, the literature on this subject is contradictory. Some studies have found no difference between genders [4, 16, 32, 35], whereas others have reported higher resilience in female students [1, 9, 36]. These discrepancies may arise from socio-cultural factors, differences in coping strategies employed by each gender, or even variations in clinical experiences [9, 16]. For instance, male students may face unique challenges in the predominantly female environment of nursing, which could affect their resilience [37].

Another interesting finding of this study was the higher resilience among students who lived “alone.” This finding contrasts with some research that associates living alone with increased feelings of loneliness and social isolation, which can reduce resilience [38]. It could be argued that the independence and personal management of affairs in solo living might lead to the strengthening of problem-solving skills and, consequently, an increase in students’ resilience, although access to social support is particularly important for this group [37]. Furthermore, while “living alone” appeared as a significant factor, this subgroup represented only 10.4% of the sample. Therefore, this finding should be interpreted cautiously as it may lack the statistical robustness of larger subgroups.

Regular nutrition and non-use of smoking/tobacco were identified as strong predictors. This finding is consistent with research literature emphasizing the positive role of proper nutrition in reducing the effects of stress and improving cognitive function [32, 34]. Similarly, research has consistently shown a negative relationship between smoking and academic resilience, as smoking is often associated with higher academic burnout and lower academic engagement [32]. Resilience can act as a moderating factor in the relationship between stress and tobacco use [32].

Favorable health status (no history of physical and especially mental illness) also emerged as a strong predictor of academic resilience among nursing students. This finding is expected and aligns with numerous studies that link good physical and mental health with higher resilience and better academic achievement [37]. Students struggling with mental health problems have fewer cognitive and emotional resources available to cope with academic pressures [17].

Satisfaction with the nursing major was another of the strongest predictors of academic resilience in the present study. Students who are satisfied with their major have higher intrinsic motivation and perceive academic challenges as more meaningful. This finding is widely supported in other studies [29, 32, 34, 37]. It appears that satisfaction with the major and academic resilience have a bidirectional and reinforcing relationship; greater satisfaction contributes to resilience, and higher resilience leads to success and, consequently, greater satisfaction with the major [37].

Finally, consistent with a vast body of research literature, regularly having support resources (such as support from family, friends, peers, and faculty) was identified as a key protective factor and a strong predictor of academic resilience. Numerous studies have shown that social support significantly increases students’ ability to cope with academic challenges and acts as a buffer against stress and burnout [3, 23, 32, 37, 39, 40]. Social support, especially from family and friends, not only directly increases resilience but can also mitigate the negative effects of stressors and help students feel less isolated when facing hardships [23, 32, 37]. Interestingly, in the present study, support from friends had the highest mean among support sources, which aligns with some studies but differs from others (which find family support more important) [32]. This may indicate the particular importance of the peer group during the university years and in the academic environment [16, 41].

In summary, the findings of this study, utilizing supplementary sources, provide a deeper understanding of the multifaceted nature of academic resilience, emphasizing that this construct is influenced by a combination of individual characteristics (health status, nutrition, smoking), social factors (support, living situation), and academic factors (satisfaction with major) [9, 24].

### Implications for practice and education

The findings of this study provide an evidence-based roadmap for educators and administrators to design targeted interventions. The significant role of individual factors—health status, balanced nutrition, and non-use of tobacco—underscores that resilience is not purely psychological. Universities must, therefore, move beyond traditional academic support. This includes integrating proactive Healthy Lifestyle workshops into curricula and providing access to nutritional counseling and robust smoking cessation programs [5, 37].

In the academic sphere, satisfaction with the major emerged as a key factor. Faculties should implement structured faculty mentorship programs to foster a positive professional identity and minimize the stress associated with clinical training. Providing realistic career counseling is essential to manage student expectations and enhance intrinsic motivation [3, 32, 39, 40].

Finally, the critical role of support resources and living situations necessitates a focus on social infrastructure. Universities must actively promote and de-stigmatize counseling services. Implementing robust peer-support programs and ensuring students living alone or in dorms are explicitly connected to university support networks are essential strategies to buffer against stressors [23, 32, 37, 42]. This comprehensive, multi-faceted approach directly addresses the identified factors and aims to cultivate a more resilient nursing workforce.

### Limitations and recommendations for future research

Despite its valuable findings, this study is subject to several methodological limitations that warrant careful consideration when interpreting the results. Primarily, the cross-sectional design restricts our ability to infer causal relationships; thus, the significant findings, although derived from regression analysis, must be interpreted cautiously as significant statistical associations. This study cannot determine the temporal direction of the relationship; for instance, does satisfaction lead to resilience, or does higher resilience foster greater satisfaction? This crucial question about causality necessitates investigation through future longitudinal designs.

Secondly, the reliance on self-report measures for all variables introduces the potential for both social desirability bias and common method variance (CMV). Furthermore, while the Academic Resilience Scale is validated, the independent variables were assessed using a researcher-developed questionnaire. Although content validity was established, the absence of full psychometric testing for this novel instrument may affect the robust reliability of some derived factors. A third limitation concerns generalizability: the sample was drawn exclusively from undergraduate nursing students in three major public universities in Tehran. Consequently, these findings may not accurately reflect the experiences of students in smaller cities, private institutions, or other health science disciplines.

Finally, certain associations, such as the observed link between resilience and the ‘living situation,’ were derived from relatively small subgroups. Such results may be statistically unstable and require cautious interpretation. For future research, we strongly recommend prioritizing longitudinal designs to track the evolution of these factors over time, incorporating qualitative methods to provide a richer, mechanistic understanding of the associations, and expanding the sampling frame to a national, multi-center level to enhance the external validity of the results.

## Conclusion

This study contributes to the existing body of knowledge by providing a comprehensive, multidimensional perspective on academic resilience among nursing students within the specific socio-cultural and educational context of Iran. Our findings reveal that academic resilience in this population is at a moderate-to-high level, yet it is intricately tied to a complex interplay of individual, academic, and social factors.

Critically, this research highlights that in the regional context of Tehran, lifestyle choices—such as balanced nutrition and non-smoking—and academic satisfaction are as pivotal as traditional social support systems in fostering resilience. By identifying a specific profile of at-risk students—particularly those residing in non-native environments or experiencing low major satisfaction—this study bridges a significant gap in regional literature. These insights offer a culturally-relevant roadmap for nursing educators and policymakers to move beyond generic support strategies. Instead, the findings advocate for the implementation of targeted, holistic interventions—ranging from lifestyle counseling to academic mentorship—designed to address the unique stressors of the Iranian nursing education environment and ultimately enhance student retention and professional competence.

## Supplementary Information

Supplementary Material 1.

## Data Availability

The datasets used and/or analyzed during the current study are available from the corresponding author on reasonable request.

## References

[1] Radhamani K, Kalaivani D. Academic resilience among students: a review of literature. Int J Res Rev. 2021;8(6):360–9.

[2] De Clercq M, Parmentier M, Van Meenen F. Fair enough?! Investigating the specific challenges of diverse university first-year students. Res Pap Educ. 2024;39(1):113–33.

[3] Panda S, Dash M, John J, Rath K, Debata A, Swain D, et al. Challenges faced by student nurses and midwives in clinical learning environment - a systematic review and meta-synthesis. Nurse Educ Today. 2021;101:104875. 33774528 10.1016/j.nedt.2021.104875

[4] Cassidy S, Mawdsley A, Langran C, Hughes L, Willis SC. A large-scale multicenter study of academic resilience and well-being in pharmacy education. Am J Pharm Educ. 2023;87(2):ajpe8998. 35338069 10.5688/ajpe8998 PMC10159510

[5] Mawdsley A, Willis SC. Academic resilience in UK pharmacy education - a pilot study applying love and break up letters methodology. BMC Med Educ. 2023;23(1):441. 37322463 10.1186/s12909-023-04380-4 PMC10268453

[6] Wei H, Dorn A, Hutto H, Webb Corbett R, Haberstroh A, Larson K. Impacts of nursing student burnout on psychological well-being and academic achievement. J Nurs Educ. 2021;60(7):369–76. 34232812 10.3928/01484834-20210616-02

[7] Ghods AA, Ebadi A, Sharif Nia H, Allen KA, Ali-Abadi T. Academic burnout in nursing students: an explanatory sequential design. Nurs Open. 2023;10(2):535–43. 36631731 10.1002/nop2.1319 PMC9834498

[8] Mendes SS, Salvi CPP, Moraes BFM, De Martino MMF. Instruments for the evaluation of stress in nursing students. J Nurs UFPE/Revista de Enfermagem UFPE. 2019;13(3):829. 10.5205/1981-8963-v13i03a236076p829-838-2019.

[9] Aryuwat P, Asp M, Lovenmark A, Radabutr M, Holmgren J. An integrative review of resilience among nursing students in the context of nursing education. Nurs Open. 2023;10(5):2793–818. 36564896 10.1002/nop2.1559 PMC10077422

[10] Alghtany S, Madhuvu A, Fooladi E, Crawford K. Assessment of academic burnout and professional self-concept in undergraduate nursing students: A cross-sectional study. J Prof Nurs. 2024;52:7–14. 38777528 10.1016/j.profnurs.2024.03.003

[11] Shin S, Hwang E. The effects of clinical practice stress and resilience on nursing students’ academic burnout. Korean Med Educ Rev. 2020;22:115–21.

[12] El Barusi AR, Kurniawati F. Systematic Literature Review: A Study of Academic Burnout among Undergraduate Students in Universities. Int J Sci Educ Cult Stud. 2024;3(1):1–18.

[13] Martin AJ, Marsh HW. Academic buoyancy: Towards an understanding of students’ everyday academic resilience. J Sch Psychol. 2008;46(1):53–83. 19083351 10.1016/j.jsp.2007.01.002

[14] Abdi M, Naghiloo MJ, Dinmohammadi M. Factors affecting the time management of graduate medical sciences students during the COVID-19 pandemic. J Med Educ Dev. 2022;15(46):22–8. 10.52547/edcj.15.46.22.

[15] Dinmohammadi M, Razaghpour H, Abdi M. Factors affecting time management ability among medical sciences students during the COVID-19 pandemic. Educ Med J. 2023. 10.21315/eimj2023.15.3.9.

[16] Latif S, Amirullah M. Students’ academic resilience profiles based on gender and cohort. Jurnal Kajian Bimbingan dan Konseling. 2024;5(4):13.

[17] Abbas A, Zahra S, Shahid S, Kashif M, Raza S. Academic Resilience, Psychological Well-Being and Suicidal Ideation among Medical and Non-Medical Students. J Health Rehabil Res. 2024;4(1):76–82.

[18] Wang F, King RB, Fu L, Chai C-S, Leung SO. Overcoming adversity: Exploring the key predictors of academic resilience in science. Int J Sci Educ. 2024;46(4):313–37.

[19] Baumgartner P, Jennifer N, Schneider P, Tamera R. A randomized controlled trial of mindfulness-based stress reduction on academic resilience and performance in college students. J Am Coll Health. 2023;71(6):1916–25. 34398703 10.1080/07448481.2021.1950728

[20] Cusack L, Smith M, Hegney D, Rees CS, Breen LJ, Witt RR, et al. Exploring environmental factors in nursing workplaces that promote psychological resilience: constructing a unified theoretical model. Front Psychol. 2016;7:600. 27242567 10.3389/fpsyg.2016.00600 PMC4866518

[21] Furtado M. Assessment of academic resilience as a non-cognitive variable in entry-level doctor of physical therapy students. J Allied Health. 2022;51(3):189–97. 36100714

[22] Hwang EH, Kim KH. Relationship between optimism, emotional intelligence, and academic resilience of nursing students: the mediating effect of self-directed learning competency. Front Public Health. 2023;11:1182689. 37275498 10.3389/fpubh.2023.1182689 PMC10234118

[23] Ye Y, Huang X, Liu Y. Social support and academic burnout among university students: a moderated mediation model. Psychol Res Behav Manag. 2021;14:335–44. 33776493 10.2147/PRBM.S300797 PMC7987309

[24] Shen Y, Feng H, Li X. Academic resilience in nusing students: a concept analysis. BMC Nurs. 2024;23(1):466. 38982439 10.1186/s12912-024-02133-2 PMC11232226

[25] Cassidy S. The Academic Resilience Scale (ARS-30): a new multidimensional construct measure. Front Psychol. 2016;7:1787. 27917137 10.3389/fpsyg.2016.01787 PMC5114237

[26] Vahedi S. Evaluation of psychometric indices of students’ academic resilience questionnaire using confirmatory factor analysis. J Psychol. 2021;99(3):373.

[27] Abou Hashish EA, Bajbeir E, Almabadi SA, Alzebali ND, Alhubishi SF. Investigating quality of life, academic resilience, and influential factors in nursing education: a mixed-methods study among nursing students. SAGE Open Nurs. 2024;10:23779608241303690. 39676903 10.1177/23779608241303690 PMC11645762

[28] Delshad ES, Nobahar M, Raiesdana N, Yarahmadi S, Saberian M. Academic resilience, moral perfectionism, and self-compassion among undergraduate nursing students: a cross-sectional, multi-center study. J Prof Nurs. 2023;46:39–44. 37188420 10.1016/j.profnurs.2023.02.006

[29] Hwang E, Shin S. Characteristics of nursing students with high levels of academic resilience: a cross-sectional study. Nurse Educ Today. 2018;71:54–9. 30245256 10.1016/j.nedt.2018.09.011

[30] Li ZS, Hasson F. Resilience, stress, and psychological well-being in nursing students: a systematic review. Nurse Educ Today. 2020;90:104440. 32353643 10.1016/j.nedt.2020.104440

[31] Tan WY, Chen JN, Lu SH, Liu CQ, Zhou Y, Luo Q, et al. Latent profiles of academic resilience in undergraduate nursing students and their association with resilience and self-efficacy. Nurse Educ Pract. 2024;77:103949. 38593563 10.1016/j.nepr.2024.103949

[32] Hamaideh S, Abu Khait A, Al Modallal H, Malak M, Masa’deh R, Hamdan-Mansour A, et al. Relationships and predictors of resilience, social support, and perceived stress among undergraduate nursing students. Open Nurs J. 2024. 10.2174/0118744346238230240103055340.

[33] Al-Hammouri MM, Rababah J, Dormans J. Exploring gender dynamics and predictors of resilience among nursing students. Nurse Educ Pract. 2024;80:104160. 39405790 10.1016/j.nepr.2024.104160

[34] Elnaem MH, Wan Salam W, Thabit AK, Mubarak N, Abou Khatwa MM, Ramatillah DL, et al. Assessment of academic resilience and its associated factors among pharmacy students in twelve countries. Am J Pharm Educ. 2024;88(5):100693. 38574997 10.1016/j.ajpe.2024.100693

[35] Van Hoek G, Portzky M, Franck E. The influence of socio-demographic factors, resilience and stress reducing activities on academic outcomes of undergraduate nursing students: a cross-sectional research study. Nurse Educ Today. 2019;72:90–6. 30463034 10.1016/j.nedt.2018.10.013

[36] Morales EE. Exceptional female students of color: academic resilience and gender in higher education. Innov High Educ. 2008;33(3):197–213.

[37] Aryuwat P, Holmgren J, Asp M, Lövenmark A, Radabutr M, Sandborgh M. Factors associated with resilience among Thai nursing students in the context of clinical education: a cross-sectional study. Educ Sci. 2024;14(1):78.

[38] Yoboua JJ. Living Conditions, Resilience and Academic Performance of Ivorian Students Girls in the Third Year. Int J Innovative Res Multidisciplinary Educ. 2024;03(08):1320–24. 10.58806/ijirme.2024.v3i8n05.

[39] Permatasari N, Ashari FR, Ismail N. Contribution of perceived social support (peer, family, and teacher) to academic resilience during COVID-19. Gold Ratio Social Sci Educ. 2021;1(1):01–12.

[40] Ozsaban A, Turan N. Resilience in nursing students: the effect of academic stress and social support. Clin Exp Health Sci. 2019;9(1):69–76.

[41] Lady GM. How can I help? Investigating the role of social supports in academic resilience for undergraduate students. Columbia: University of South Carolina; 2021.

[42] Aghamohammadi F, Saed O, Dinmohammadi M. Factors affecting the resilience of Iranian nurses during the COVID-19 pandemic. J Client Cent Nurs Care. 2023;9(3):223–30. 10.32598/JCCNC.9.3.451.

