# Supplementary material for: Factors associated with academic resilience in nursing students: the role of individual, academic, and social factors
Source: BMC Med Educ. 2026 Feb 27;26:555. doi: 10.1186/s12909-026-08875-8 (PMC13049952; doi:10.1186/s12909-026-08875-8)
Supplement: Supplementary file 1 — Supplementary Material 1. [file 12909_2026_8875_MOESM1_ESM.pdf]

Supplementary File 1: Individual, Academic, and Social Factors Questionnaire

This questionnaire was developed by the researchers for the study titled **"Predictors of Academic Resilience in Nursing Students: The Role of Individual, Academic, and Social Factors"** and is presented here as required by the journal.

Section A: Individual Factors

1. **Age:** .... years
2. **Gender:**
   - ( ) Male
   - ( ) Female
3. **Marital Status:**
   - ( ) Single
   - ( ) Married
4. **Native Status:**
   - ( ) Native (Tehran)
   - ( ) Non-Native (Other Cities)
5. **Accommodation Type:**
   - ( ) Living with family at home
   - ( ) Dormitory
   - ( ) Alone (outside home/university environment)
6. **Sleep Status (per 24 hours):**
   - ( ) Sufficient/Regular sleep
   - ( ) Oversleeping
   - ( ) Insufficient sleep
7. **Exercise:**
   - ( ) Yes; Regularly
   - ( ) Yes; Occasionally
   - ( ) No
8. **Balanced Nutrition:**
   - ( ) Yes; Regularly
   - ( ) Yes; Occasionally
   - ( ) No
9. **Religious Practices:**
   - ( ) Yes; Regularly
   - ( ) Yes; Occasionally
   - ( ) No
10. **Smoking/Hookah Use:**
    - ( ) Yes; Regularly

- ( ) Yes; Occasionally
- ( ) No

11. **Social Media Usage:**
    - ( ) Yes; Regularly
    - ( ) Yes; Occasionally
    - ( ) No
12. **Current Health Status:**
    - ( ) Complete physical and mental health
    - ( ) History of physical illness
    - ( ) History of mental illness

Section B: Academic Factors

13. **Academic Year:**
    - ( ) First Year (Semesters 1&2)
    - ( ) Second Year (Semesters 3&4)
    - ( ) Third Year (Semesters 5&6)
    - ( ) Fourth Year (Semesters 7&8)
14. **Cumulative GPA (Latest Semesters):** ....
15. **Satisfaction with Nursing Major:**
    - ( ) Yes
    - ( ) No
16. **Satisfaction with the University of Study:**
    - ( ) Yes
    - ( ) No
17. **Are you considering continuing education in higher degrees (Master's and PhD)?**
    - ( ) Yes
    - ( ) No
18. **Are you considering withdrawing from the Nursing Major?**
    - ( ) Yes
    - ( ) No
19. **Are you considering emigration after graduation?**
    - ( ) Yes
    - ( ) No

Section C: Social Factors

20. **Employment Status:**
    - ( ) Employed
    - ( ) Unemployed
21. **Income Status:**
    - ( ) Yes (Have income)

    - ( ) No (Do not have income)
22. **Peer Relationships:**
    - ( ) Yes; Regularly
    - ( ) Yes; Occasionally
    - ( ) No
23. **Participation in Extracurricular Activities (Cultural, sports, etc.):**
    - ( ) Yes; Regularly
    - ( ) Yes; Occasionally
    - ( ) No
24. **Benefit from Support Resources (Financial family support, having close friends, etc.):**
    - ( ) Yes; Regularly
    - ( ) Yes; Occasionally
    - ( ) No
